# Food Safety and Cross-Contamination of Gluten-Free Products: A Narrative Review

**DOI:** 10.3390/nu13072244

**Published:** 2021-06-29

**Authors:** Herbert Wieser, Verónica Segura, Ángela Ruiz-Carnicer, Carolina Sousa, Isabel Comino

**Affiliations:** 1 Independent Researcher, 85354 Freising, Germany; h.wieser2@gmx.de; 2 Department of Microbiology and Parasitology, Faculty of Pharmacy, University of Seville, 41012 Seville, Spain; vsegura@us.es (V.S.); acarnicer@us.es (Á.R.-C.); csoumar@us.es (C.S.)

**Keywords:** celiac disease, gluten cross-contaminations, dietary adherence, gluten-free diet, vital gluten, oat, hidden gluten

## Abstract

A gluten-free diet (GFD) is currently the only effective treatment for celiac disease (CD); an individual’s daily intake of gluten should not exceed 10 mg. However, it is difficult to maintain a strict oral diet for life and at least one-third of patients with CD are exposed to gluten, despite their best efforts at dietary modifications. It has been demonstrated that both natural and certified gluten-free foods can be heavily contaminated with gluten well above the commonly accepted threshold of 20 mg/kg. Moreover, meals from food services such as restaurants, workplaces, and schools remain a significant risk for inadvertent gluten exposure. Other possible sources of gluten are non-certified oat products, numerous composite foods, medications, and cosmetics that unexpectedly contain “hidden” vital gluten, a proteinaceous by-product of wheat starch production. A number of immunochemical assays are commercially available worldwide to detect gluten. Each method has specific features, such as format, sample extraction buffers, extraction time and temperature, characteristics of the antibodies, recognition epitope, and the reference material used for calibration. Due to these differences and a lack of official reference material, the results of gluten quantitation may deviate systematically. In conclusion, incorrect gluten quantitation, improper product labeling, and poor consumer awareness, which results in the inadvertent intake of relatively high amounts of gluten, can be factors that compromise the health of patients with CD.

## 1. Introduction

The consumption of gluten proteins drives adverse reactions in predisposed individuals who suffer from celiac disease (CD), wheat allergies, non-celiac gluten sensitivity, dermatitis herpetiformis, or gluten ataxia [1,2,3]. CD is one of the most frequent hypersensitivities, affecting around 1% of the world’s population [4]. It is an immune-mediated systemic disorder caused by ingestion of gluten in genetically susceptible individuals and is based on a variable combination of intestinal and extra-intestinal signs and symptoms that are specific to CD antibodies, HLA-DQ2/8 haplotypes, and enteropathy [5,6]. 

A strict gluten-free diet (GFD) is currently the only safe and efficient therapy for patients with CD [7,8], which implies that all gluten-containing foods and meals, produced from wheat, rye, barley, and some varieties of oats, must be completely excluded from the diet. Nevertheless, such a diet is difficult to follow because of the unintended contamination of “gluten-free” products, improper labeling, social constraints, and ubiquity of gluten proteins in raw or cooked food and pharmaceuticals. Thus, accidental gluten encounters are likely. Most patients with CD can safely tolerate a daily cross-contamination of approximately 10 mg gluten, or 500 g of food containing 20 mg/kg of gluten. However, there is a tremendous degree of variability within this population, and some patients may have worsening histological changes with very low daily gluten exposure [9,10]. 

People needing to follow a GFD may consume gluten-free foods from two categories. First, they are allowed to eat a wide range of naturally gluten-free foods such as meat, fish, milk products, vegetables, nuts, and fruits. Second, patients may consume dietetic gluten-free products, i.e., alternatives to traditional gluten-containing foods, which are labeled as gluten-free. These are made from cereals that do not contain gluten such as rice, corn, sorghum, and millet, or pseudocereals such as amaranth, buckwheat, and quinoa [11]. Recent advances in the formulation of cereal-based gluten-free products by utilizing alternate ingredients and processing techniques have been summarized by Rai et al. [12]. Definitions, thresholds, and labeling of dietetic gluten-free foods have been specified in international and national regulations. According to the “Codex Alimentarius Standard for Gluten-Free Foods” [13], gluten-free dietetic foods labeled gluten-free should not exceed 20 mg gluten per kg food when sold to consumers [13]. 

Due to gluten contamination, many inherently gluten-free products (derived from corn, rice, millet, etc.,) cannot be consumed by patients with CD. These products, if misbranded as “gluten-free” and used by the patients with CD, could result in a recurrence of symptoms. Contamination of gluten-free foods with gluten-containing material can occur at many stages of food production, from the fields, farms, mills, and factories, as well as handcraft enterprises, restaurants, and households [14,15]. Until the 1970s, sensitive and accurate quantitation of gluten contamination was not possible, and patients with CD were constantly at risk of inadvertent intake of high amounts of gluten [16]. The picture has clearly improved in recent decades, most likely due to the development of immunochemical methods such as the Enzyme-Linked ImmunoSorbent Assay (ELISA), for gluten detection and the worldwide implementation of the 20 mg/kg maximum threshold of gluten contamination established by the “Codex Alimentarius 118–179” in 2008 [13,17,18]. However, patients with CD are still confronted with foods that are contaminated by gluten above the threshold of 20 mg/kg.

The major aim of this review is to provide insight into the frequent occurrence of gluten in naturally or certified gluten-free foods, as well as the safety of oats as part a GFD and the problem with hidden gluten. In addition, we examine the immunochemical and non-immunochemical methods currently available for the detection of gluten.

## 2. Methods

PubMed database searches were performed for articles published until March 2021. The search terms used included “c(o)eliac disease, gluten-free diet”, “gluten, contamination”, “gluten, oats”, “gluten, quantitation”, and “gluten, quantification”. References of included full-text articles were scrutinized for additional studies. We included published articles and review reporting on gluten contamination in gluten-free foods. We excluded publications that did not focus on the aim of this review. Only English publications were selected during the search. Case reports, commentaries, conference papers, and letters were excluded. Retrieved manuscripts were reviewed by the authors, and the data were extracted and described.

## 3. Gluten Contamination in Gluten-Free Foods

### 3.1. Naturally and Certified Gluten-Free Foods

In the 1980s and 1990s, newly developed ELISAs, mainly optimized for the detection of wheat gliadins, enabled the sensitive detection of gluten contamination in gluten-free products. At that time, the analytic results demonstrated that raw materials, naturally gluten-free by origin and used for the production of gluten-free products such as rice, buckwheat, corn, or millet flours, were contaminated with wheat up to 3000 mg gliadin/kg [19]. Later, the levels of gluten contamination in gluten-free products were found to be distinctly lower; however, contamination is still a problem, as shown in the following examples (Table 1).

Twenty-two naturally gluten-free grains, seeds, and flours were purchased in the United States and tested in duplicate for gluten contamination [31]. Thirteen samples (59%) contained less than the limit of quantitation (5 mg/kg), and nine samples (41%) contained gluten levels ranging from 8.5 to 2925 mg/kg. Seven samples (32%) exceeded 20 mg/kg and could not be considered gluten-free. In another market survey in the United States, different gluten-free-labeled foods (*n* = 275) and non-labeled foods (without wheat/rye/barley on the ingredient label; *n* = 186) were analyzed for gluten [24]. A total of 1.1% of gluten-free-labeled foods had gluten contents >20 mg/kg. Among the non-labeled naturally gluten-free foods, 19% had >20 mg/kg of gluten, of which 10% had >100 mg/kg. The investigation of 78 different certified gluten-free foods, offered in the United States, revealed that 61% of the samples contained less gluten than the limit of quantitation (10 mg/kg), and 18% contained between 10 and 20 mg/kg [22]. However, 21% had gluten levels above 20 mg/kg, ranging from 21 to 61 mg/kg. In particular, five of eight labeled breakfast cereal samples showed gluten contents >20 mg/kg.

A large Canadian investigation of naturally gluten-free ingredients, such as flours and starches, showed that 61 of the 640 samples (9.5%) were contaminated with gluten above 20 mg/kg [32]. The largest and most consistent mean contamination came from soy (902 mg/kg), millet (272 mg/kg), and buckwheat (153 mg/kg). An examination of gluten-free products from 25 bakeries in Brasilia revealed that 28 of 130 samples (22%) were contaminated with gluten above 20 mg/kg [35]. This finding was even more concerning considering that 16 bakeries (64%) sold at least one product contaminated with gluten. Only nine establishments (36%) had no gluten-contaminated products in their assortments.

A total of 200 commercially available naturally or certified gluten-free products were randomly collected from different Italian supermarkets and analyzed [34]. Gluten levels were lower than 10 mg/kg in 173 products (87%), between 10 and 20 mg/kg in 9 samples (4.5%), and higher than 20 mg/kg in 18 samples (9%). Contaminated foodstuffs (gluten >20 mg/kg) most commonly belonged to oat-, buckwheat-, and lentil-based items. Naturally gluten-free products were at a significantly higher risk of contamination than products certified as gluten-free. To study the evolution of gluten contamination over time, a total of 3141 cereal-based gluten-free foodstuffs, sold in Spain from 1998 to 2016, were consecutively analyzed [25]. Products were divided into eight categories: flours, breakfast cereals/bars, bakery, pasta, breads, dough, snacks, and yeast. Overall, gluten exceeding 20 mg/kg was detected in 371 samples (12%), with breakfast cereals/bars being the most contaminated group (Table 1). Data obtained on the analyzed products demonstrated that cereal-based gluten-free foods have become safer over time, but gluten contamination remains a problem. One of the few pleasing findings regarding gluten contamination was the reported selection of European foods labeled as gluten-free [21]. A total of 205 representative products among six food groups (bread, pasta, pastry, biscuits, pizza, and breakfast cereals), purchased from markets in Italy, Spain, Germany, and Norway, were investigated. The gluten content ranged between <5 and 28 mg/kg, and only one sample (0.5%) had a gluten concentration above 20 mg/kg.

Miscellaneous gluten-free foods were investigated for gluten contamination to evaluate the situation in Turkey [26]. A total of 200 samples from eight product categories (snack, pasta, bread, cookie, cracker, farina, traditional, and others), manufactured using seven ingredient categories (cereal mixture, buckwheat, corn, rice, locust bean, potato, and others), were analyzed. A significant proportion of the samples (17.5%) were contaminated with gluten, and therefore unacceptable in terms of being called gluten-free. The results pointed to buckwheat as the main cause of this contamination. To evaluate gluten contamination in Lebanon, 173 gluten-free food samples were analyzed over a 2-year period [33]. In 10 samples (6%), the quantity of gluten exceeded 20 mg/kg (Table 1). Eight of the contaminated samples were locally manufactured and based on wheat starch. To assess the gluten content of labeled and naturally gluten-free grain products from markets in Southern India, different “gluten-free” breakfast products, flours, and batters (*n* = 160) were evaluated [30]. Nearly 36% of the products made from naturally gluten-free grains and 10% of gluten-free-labeled products were found to contain >20 mg/kg gluten.

The ingestion of purified wheat starch as a constituent of gluten-free products is considered safe in many countries, but uncertainties about residual gluten amounts remain. Due to the generally low ratio of gliadins to glutenins in starch, gluten levels determined by ELISA are likely to be underestimated. In comparative analyses of gluten content, eight gluten-free starch samples were analyzed by R5 ELISA (gluten = gliadins × 2) and a chromatographic control method (gluten = gliadins + glutenins) [36]. According to ELISA testing (12–30 mg/kg), only two samples were not gluten-free (21 and 30 mg/kg). In contrast, all eight samples had gluten contents >20 mg/kg (38–69 mg/kg) when gliadins and glutenins were accounted for in chromatographic analysis.

In a recent systematic review, 24 cross-sectional studies were analyzed to evaluate the prevalence of gluten contamination in gluten-free industrial and non-industrial products. The authors evaluated the methodological quality of the included studies using criteria from a Meta-analysis of Statistics Assessment and Review Instrument (MASTARI). In total, 95.83% (*n* = 23) of the studies presented positive results for contamination (contained gluten above 20 mg/kg). In industrial food products, studies showed a contamination prevalence of 13.2% (95% CI: 10.8–15.7%). In non-industrial food products, studies showed a contamination prevalence of 41.5% (95% CI: 16.6–66.4%). Despite the non-industrial products presenting a higher contamination prevalence than the industrial products, the difference was not significant (*p* = 0.072). The findings indicated cross-contamination in industrial and non-industrial products [37].

In conclusion, most studies on gluten contents in naturally or certified gluten-free foods revealed relatively high rates of contamination, ranging from 0.5% to 36% of the analyzed samples (Table 1). Contaminated naturally gluten-free products appear to be a higher health risk than certified products for patients with CD. Altogether, both naturally and labeled gluten-free foods do not guaranty safety for patients with CD, and gluten contamination is an important cause of inadvertent non-adherence to a GFD.

### 3.2. Products from Food Services

Eating at restaurants, workplaces, schools, and home (own or other people’s) remains a distinctive risk for inadvertent gluten exposure. In a systematic review, 24 international studies were used to investigate gluten contamination (>20 mg/kg) in gluten-free products from food services and industries [37]. The statistical meta-analysis resulted in a mean contamination prevalence of 42% (17–66%) in certified products offered by food services. Furthermore, a mean contamination of 13% (11–16%) was detected in industrial food products labeled as gluten-free. The examination of gluten-free-labeled foods, offered in a number of restaurants across the United States, resulted in surprisingly high rates of gluten contamination [29]. A total of 5624 tests were performed by 804 users equipped with a portable gluten detection device (Nima, Nima Labs, Inc., San Francisco, CA, USA). Data were collected during an 18-month period and sorted by food items and regions. Gluten above 20 mg/kg was detected in 32% of products labeled as gluten-free (Table 1). Rates of gluten detection differed by meal, with 27.2% at breakfast and 34.0% at dinner. Gluten-free labeled pizza and pasta were most likely to test positive for gluten, with gluten detected in 53.2% of pizza samples and 50.8% of pasta samples.

The evaluation of gluten content in gluten-free food on request in restaurants in Ireland revealed that the majority of attempts to purchase a gluten-free meal were successful [20]. However, some 10% of all samples contained gluten above 20 mg/kg: 2.7% between 21 and 100 mg/kg and 7.7% above 100 mg/kg, and two unsatisfactory samples were purchased from so-called celiac-friendly restaurants (Table 1). In a study from Brazil, common beans were collected from different self-service restaurants in Brasilia and later analyzed for gluten content [23]. The results revealed that 16% of the samples were contaminated with gluten above 20 mg/kg and almost 45% of the restaurants had gluten contamination in beans on at least one of the days tested (Table 1).

To determine the rate of gluten contamination in typical Brazilian lunch meals, traditionally gluten-free, a total of 180 dishes were purchased from 60 food services in the Federal District Brazil [28]. They were visited at lunch time, and the dishes were chosen randomly. Three different food items were collected for gluten analysis in each food service. Fortunately, only 3% of dish samples were contaminated with gluten (Table 1), and only 7% of food services displayed at least one contaminated food. Thus, traditional Brazilian dishes, made from naturally gluten-free materials, appeared to be safe for patients with CD. Another positive example was found in Italy: all pizzas and cooked dough bases (*n* = 56), produced at certified take-away pizza restaurants in the Turin metropolitan area, were gluten-free (<20 mg/kg) [27]. Thus, attention to and compliance with good manufacturing practices, a requisite for obtaining gluten-free certification for restaurants in Italy, have a positive effect on the production of gluten-free products.

A quantitative assessment of gluten cross-contact in the school environment for children with CD measured the gluten transfer from school activities to gluten-free foods that a child may eat afterwards [38]. Five experiments were used to identify potential gluten transfer to gluten-free bread in classrooms using sensory tables: Play-Doh, baking projects, papier-mâché, dry pasta, and cooked pasta. After activities, slices of gluten-free bread were rubbed on participant’s hands and table surfaces and gluten levels were determined. The potential for gluten exposure was found to be high (>20 mg/kg) for papier-mâché, baking projects, and cooked pasta.

Meals may be contaminated not only in food services but also at home. Gluten-free meals should always be prepared, stored, and handled separately from gluten-containing meals. If separate areas are not available, preparing a gluten-free meal before other meals is recommended. However, the need for extra cooking is frequently seen as a problem for maintaining a GFD.

In conclusion, gluten-free products from food services hold a considerable risk for gluten contamination. Patients with CD are advised to check allergen lists, according to Codex Standard 1- 985, and/or to ask staff for information. Food services should make efforts to minimize the risk of cross-contamination in food (Table 1). This would create a more reliable environment for patients with CD who need to eat when away from home. Furthermore, future research should focus on identifying inappropriate procedures that cause gluten-contamination and should propose new strategies to overcome this issue.

### 3.3. Oats

The necessity of excluding oats from the diet of patients with CD is still a matter of discussion. An update of the ongoing debate on oats, and the pros and cons of using oats in a GFD, was reviewed by Cohen et al. [39] and Hoffmanova et al. [40]. Most clinical studies have reported that moderate amounts of pure oats are well-tolerated by most patients with CD, and only a small number of patients (probably less than 1%) experience harmful effects from oat consumption. Therefore, in many countries, oats are recommended to be included as part of a GFD. The high contents of beneficial compounds such as dietary fiber, unsaturated fatty acids, and antioxidants make oats an attractive component of a GFD. However, oat products can only be tolerated if they are free from wheat, rye, and barley. Pure oats must meet the legislative criteria for gluten-free foods, i.e., the content of gluten from wheat, rye, and barley in the end-product must be less than 20 mg/kg.

The fact that oats are often processed on the same production line as wheat, rye, or barley is a major cause of gluten contamination. Therefore, it is not surprising that commercial oat supply can be heavily contaminated with gluten-containing grains. However, recognition and measurement of gluten contamination in oat products with ELISA kits are still a problem. At AOAC (Association of Official Analytical Collaboration) International, a stakeholder panel convened and agreed upon standard requirements for the quantitation of total wheat, rye, and barley gluten in oat products by ELISA [41]. The defined method acceptance criteria were 5–15 mg/kg of gluten as the analytical range, limits of detection and quantitation below 5 mg/kg of gluten, and 50–200% recovery. The rather wide recovery range was chosen due to the lack of homogeneity inherent in oat samples and different ELISA antibody responses to gluten proteins from wheat, rye, and barley.

If oats are used in a GFD, it is recommended that contamination with wheat, rye, and barley be assessed by a stepwise “test-all-positives” methodology [42]. Oat groats are split into 75 g samples and ground. Afterwards, a 15 g portion is analyzed using a sandwich ELISA. A result of >20 mg/kg disqualifies the production lot, while a result of <20 mg/kg triggers complete analysis of the remaining 60 g of ground sample, which is analyzed in 15 g portions. If all five 15 g tests are <20 mg/kg, the lot can be approved.

One of the first studies on gluten contamination in oats, conducted in Spain in 2006, evaluated 108 oat samples (e.g., rolled oats, oat flakes, and flours) collected from Europe, the United States, and Canada [43]. Three quarters of the samples were contaminated with more than 20 mg/kg of gluten, with a variation of up to 8000 mg/kg. A pilot study on gluten contamination in grains, seeds, and flours in the United States, which included rolled or steel-cut oat samples into the investigation [31], found 9 out of 12 containers, representing four different lots of each of three separate brands (Quaker, Country Choice, and McCann’s), had gluten levels ranging from 23 to 1807 mg/kg. Another study, performed in 2011, demonstrated that approximately 88% of 133 Canadian commercial oat samples were contaminated with gluten above 20 mg/kg, and there were no differences between the oat types tested [44]. Among grain-based food products purchased from markets in Southern India, 85% of the oat samples were contaminated with gluten in amounts up to 1830 mg/kg [30].

However, differences in the type of oat grain, oat purity, study design, as well as the specifications for gluten-free products in different countries, are some reasons why the current studies have not clearly established whether oats can be safely consumed by all patients with CD. These apparent contradictions might be explained by the fact that the oat varieties used in the diverse studies were different in terms of their prolamin genes, protein amino acid sequences, and the immunoreactivities of their toxic prolamins [45,46,47,48]. Even so, some pure oats cultivars have significant reactivity with the most used monoclonal antibodies R5, G12 [48,49,50,51]. Some celiac T-cell activating sequences from oats have been identified [52,53,54], and some oat varieties have elicited early inflammatory events typical of CD [47]. Despite this evidence, it is still commonly believed that there is no reactivity in pure oats.

In conclusion, while the inclusion of oats in a GFD might be beneficial due to their nutritional and health benefits, the source of the oats used and the cultivar selected are important factors to be considered. It is extremely important to remember that in vitro studies have shown that the immunogenicity of oats varies depending on the cultivar used. In any case, it seems that lack of reactivity with immune assays (R5, G12) may guarantee the absence of toxic gluten regardless of source-from oats itself or from wheat or barley contamination. These factors must also be taken into account when developing food safety regulations, labeling oat-containing products as gluten-free, and designing clinical trials to study the effect of oats in patients with CD for evidence of adverse reactions.

### 3.4. Hidden Gluten

Patients with CD should be aware of numerous composite foods, medications, and cosmetics that contain “hidden” sources of so-called vital gluten (VG), a by-product of wheat starch production. Therefore, patients are advised to check the ingredients labeled on prepacked products or to obtain information about unpacked foods. Prepacked foods should conform to the regulations of the Codex Standard 1-1985. To protect sensitive consumers from harmful allergic symptoms, the “General Standard for the Labeling of Prepacked Foods” states that the following foods and ingredients, which contain proteins known to cause allergies and other types of hypersensitivities, should always be declared (the top eight food allergens): cereals containing gluten, crustaceans, eggs, fish, peanuts, soybeans, milk, and tree nuts.

However, clear food labeling is not a requirement in all countries. Moreover, a number of patients with CD are not motivated to study the label when they buy prepacked foodstuffs made from naturally gluten-free materials. Likewise, patients usually do not ask for information on unpacked foods that consist of naturally gluten-free components. Thus, hidden VG is one of the main initiators of inadvertent breaks with a GFD. VG is typically added to food in the range of 1–3 g/100 g of dry mass. The water-binding and thickening properties of VG are used to improve the quality of ice cream, coffee creamer, instant pudding, soups, sauces, ketchup, marinades, and dressings. Due to the outbreak of bovine spongiform encephalopathy (BSE) and the subsequent efforts to replace gelatine, VG has found new applications in the production of some special foods such as chewing gum, chew candies, and fruit chews [55].

The properties of VG help to bind vitamin/mineral components to fortified corn flakes, puffed rice, or grain berries. Another application is the coating of dry roasted nuts with VG, which enables the adhesion of salt and other seasonings. VG is also applied as an additive in the production of soy sauce. The binding, film-forming, and thermosetting characteristics of hydrated VG are the basis for various applications in the manufacture of meat products. It is effective in binding meat chunks to form special products such as textured meat, canned hams, and poultry rolls. VG is also useful as a protein binder in sausages and other meat emulsion products. The incorporation of about 2% VG into surimi enhances gel strength and reduces the development of an undesirable rubbery texture after frozen storage.

An increasing number of people opting for a vegetarian or vegan diet has increased the demand for substitutes of animal products that are often produced with the help of VG. Gelatin, used as a thickening and gelling agent, is frequently replaced by VG. The viscoelastic properties of hydrated VGs are exploited for the production of synthetic cheese, with the characteristic texture and sensory properties of natural cheese. The production of seafood analogues is another field of VG utilization.

Gluten is introduced into numerous medications, mostly through the use of wheat starch as a filling agent. Conventional wheat starch contains approximately 3000–4000 mg gluten/kg and, thus, can cause significant gluten contamination in medical products. Apart from medications, wheat starch plays a role in the production of dialysis solutions, enteral nutrition, and even as a substitute for blood plasma. In 2011, the US Food and Drug Administration (FDA) solicited information and public comments on the use of gluten in drug products, but did not include gluten labeling of drugs. Thus, patients with CD do not know whether a product is gluten-free or not, unless it is labeled as such. Therefore, all drug products should be made gluten-free, because there are many alternatives to gluten-containing materials, such as starch, that can be used as excipients during their formulation [56].

VG is frequently added to oral hygiene and cosmetic products such as toothpastes, mouthwashes, and lipsticks. Of 66 items collected from an Italian market, 62 samples were found to be gluten-free (<20 mg/kg), while three toothpastes (21–35 mg/kg) and one lipstick (27 mg/kg) showed a gluten level above 20 mg/kg [57].

In conclusion, hidden gluten in naturally gluten-free foods and drugs is a major contributor to inadvertent gluten intake. In particular, gluten being added to certain products, inadequate labeling, and poor knowledge on the part of consumers are important factors that compromise the health of patients with CD.

## 4. Analytical Methods to Detect Gluten

Currently, patients with CD are confronted with uncertainties in gluten analysis and, accordingly, may run the risk of inadvertent gluten intake due to inaccurately determined gluten levels. Many methods have been developed for the detection of prolamins, including polymerase chain reaction (PCR), liquid chromatography–mass spectrometry (LC–MS), and immunological methods based on anti-gluten peptide antibodies. The use of LC–MS is difficult because of its cost and technical performance, as well as the complexity of the sample which contains many different peptides. Accordingly, immunoassays such as ELISAs and lateral flow devices (LFD) have been the methods of choice in the food industry to certify gluten-free food because of their combination of specificity, sensitivity, simplicity, and cost effectiveness (Table 2). In recent years, methods have been developed for use by celiac patients themselves. Specifically, Nima™ and EZ Glutent^TM^ LFD have been developed to integrate food processing and gluten detection in a portable device that is available for consumer use [58,59].

A number of gluten-specific ELISA, LFD, and PCR kits are commercially available worldwide (Table 3). Each kit has specific features, such as format (sandwich, competitive and LDF), sample extraction buffers, extraction time and temperature, characteristics of the antibodies, and target analysts, as well as the reference material used for calibration. Due to these differences and a lack of official reference material, the results of gluten quantitation may deviate systematically; a number of publications have highlighted that routine ELISA methods do not provide equivalent results for gluten content [60,61,62].

Assays show high variability in specificity that corresponds to the type of cereal, the species, and the variety, as well as the composition of gluten protein types. For example, the evaluation of the five most frequently used ELISA kits showed high variability towards different cereals and gluten protein types [63,64]. Similarly, the determination of gluten in different cultivars of common wheat, spelt, durum wheat, emmer, and einkorn, using three ELISA kits, showed clear differences between kits [65]. The comparison of five ELISA kits containing two polyclonal antibodies and three monoclonal antibodies revealed that wheat prolamins (gliadins) were detected accurately by all tested antibodies, but high variability was observed for rye and barley prolamins [64]. The gluten content (sum of prolamins and glutelins) was either overestimated up to six times (rye) or underestimated up to seven times (barley). Avenins, the gluten proteins of oats, remain a challenge in terms of detection and quantitation because most antibodies used in ELISA do not react with avenins, except monoclonal antibodies G12 and R5 [45,46,47,48].

Further problems have been found in determining the gluten content of foods with different matrices. A set of 14 ELISA kits for gluten detection was used to analyze gluten levels in a series of relevant food matrices that varied in complexity [60]. The results demonstrated that there was no single ELISA kit that could accurately detect and quantify gluten in all different matrices. Additional difficulties may be caused by food processing that impairs the detection of gluten, such as heat treatment, extrusion, or fermentation. Accurate quantitation of gluten by antibody-based methods in fermented foods such as beer, baby food, or soy sauce is a particular challenge. The reduced recovery of gluten by ELISA after enzymatic partial hydrolysis of gluten proteins is a well-studied effect. For example, the quantitation of a peptic/tryptic gliadin hydrolysate by a competitive ELISA resulted in 56% recovery compared to the starting gliadin material [66], although toxicity to patients with CD was sustained after peptic/tryptic digestion [67]. It is uncertain whether ELISA, even in a competitive format, is an appropriate method for identifying partially hydrolyzed gluten. Potential diversity in the generation of sequences, relative abundance, and extension of the resultant gluten peptides is almost limitless [68,69]. The estimation of gluten equivalence in hydrolyzed gluten samples is thus a challenge. Firstly, peptides may have only one epitope per molecule. The best approach to measure the immunogenicity level of a beer, for example, is to use two antibodies that are capable of recognizing the peptides of gluten that comprise most of the immunotoxic response of these proteins. These antibodies must recognize sequences that do not overlap one another. Therefore, the difference in estimations between different antibody-based methods could be appreciated more in hydrolyzed food or beverages because of differential resistance of the corresponding epitopes observed [70]. Thus, the concept of gluten content should ideally be changed to gluten immunogenic peptides in beer and other hydrolyzed food, as gluten proteins are actually hydrolyzed and peptides are what remain. A potential risk is that absorption is faster because it may not need digestion in the stomach and intestine to have immunogenic peptides that may be rapidly absorbed.

Moreover, the reactivity and number of immunogenic peptide sequences may vary among different wheat [71,72,73] and barley [74] varieties. All flours from hexaploid wheats (common wheat and spelt) studied by Schalk et al. [73] contained the immunogenic 33-mer peptide. In contrast, the 33-mer was absent (<limit of detection) from tetra- and diploid species (durum wheat, emmer, einkorn), most likely because of the absence of the D-genome, which encodes α-gliadins. In Comino et al. [75], eight different barley cultivars were analyzed by G12 ELISA, revealing 25-fold differences in reactivity between the most and the least reactive barley cultivars. Three of those cultivars were analyzed by T-cell activation, and the hierarchy of immunogenicity with T-cells isolated from peripheral blood was consistent with the reactivity of the barley kernels.

Most ELISA methods are based on quantifying the prolamin fraction and not the glutelin fraction. To account for this bias, the determined prolamin content is usually multiplied by a factor of two to obtain the gluten content, assuming a prolamin/glutelin ratio of one (Codex Standard 118–179). However, the true ratios are highly variable, ranging from 0.2 in wheat starch to 13.9 in einkorn flour [76]. Consequently, the gluten content of wheat starch tends to be underestimated when the prolamin × 2 for calculation is applied, as shown by Scherf et al. [77]. Comparative analysis of gluten content in eight starch samples, labeled as gluten-free by R5 ELISA (prolamin × 2) and a chromatographic control method (sum of prolamins and glutelins), revealed highly different mean values (15 vs. 54 mg/kg). Moreover, gluten analysis of 30 wheat starch samples (14 declared as gluten-free) with seven commercial ELISA kits resulted in up to six different values per sample [77].

In conclusion, an ideal antibody for gluten analysis should not only be a reliable indicator of the presence of prolamins from cereal species known to be toxic to patients with CD, but also should recognize the specific intramolecular regions responsible for such immunotoxicity; as such, it would not underestimate the potential immunogenicity of certain hydrolytic materials [61]. Thus, the reactivity of monoclonal antibodies used in the detection of gluten content could provide different estimations that should be verified with real immunogenicity in human samples. Moreover, ELISA testing is still the most useful method for gluten quantitation and, despite the variability between tests, it provides acceptable results regarding the raw materials used in gluten-free food production. Problems exist in analyzing gluten contamination in wheat starch and processed foods, e.g., heat-treated or fermented foods, and these require further research and development.

## 5. Conclusions

Most studies on the gluten contents of naturally or certified gluten-free foods reveal relatively high rates of contamination, and contaminated naturally gluten-free products appear to be a higher health risk than certified products for patients with CD. Thus, both naturally and labeled gluten-free foods do not guarantee safety for patients with CD, and gluten contamination is an important cause of inadvertent non-adherence to a GFD. Oats could be included in a GFD, provided that the absence of toxic gluten from the oats themselves, or from contamination by wheat, barley, or rye, is guaranteed. Additionally, gluten-free products from food services represent a considerable risk for gluten contamination. Patients with CD should be aware of numerous composite foods, medications, and cosmetics that contain “hidden” gluten that is used as an additive to improve the properties of gluten-free foods. Many methods have been developed for the detection of gluten proteins, including ELISA, PCR, LFD, and LC/MS. ELISA testing is still the most useful method for gluten quantitation and, despite the variability between tests, it provides acceptable results. Problems exist in analyzing gluten contamination in wheat starch and processed foods, e.g., heat-treated or fermented foods, and these require further research and development.

## Figures and Tables

**Table 1:** Studies on the rate of gluten contamination in natural and certified gluten-free foods, as well as food service products.

Type of Products	Study Country	*n*	Percentage of Food Containing >20 mg/kg of Gluten	References
	Ireland	260	10%	McIntosh et al., 2011 [20]
Gluten-free-labeled products	Italy, Spain, Germany, and Norway	205	0.5%	Gibert et al., 2013 [21]
	United States	78	21%	Lee et al., 2014 [22]
	Brazil	20	16%	Oliveira et al., 2014 [23]
	United States	275	1.1%	Sharma et al., 2015 [24]
	Spain	3141	12%	Bustamante et al., 2017 [25]
	Turkey	200	17.5%	Atasoy et al., 2017 [26]
	Italy	56	0%	Bianchi et al., 2018 [27]
	Brazil	180	3%	Farage et al., 2019 [28]
	United States	5624	32%	Lerner et al., 2019 [29]
	Indian	160	36%	Raju et al., 2020 [30]
Naturally gluten-free products	United States	22	32%	Thomson et al., 2010 [31]
	Canada	640	9.5%	Koerner et al., 2013b [32]
	United States	186	19%	Sharma et al., 2015 [24]
	Indian	160	10%	Raju et al., 2020 [30]
Naturally or certified gluten-free products	Lebanon	173	6%	Hassan et al., 2017 [33]
	Italy	200	9%	Verma et al., 2017 [34]
	Brazil	130	22%	Farage et al., 2017 [35]

**Table 2:** Analytical techniques for the detection and quantification of gluten.

	Strengths	Weak Points
**ELISA immunoenzyme assay**	Simple to perform, fast, inex-pensive, high sensitivity, does not produce cross-reactions.	False negatives can occur when proteins are denatured by changes in pressure, temperature, or salt concentration.
**POLYMERASE CHAIN REACTION (PCR)**	Very high sensitivity in the detection of DNA, allows one to identify the species from which the gluten comes, useful to identify the origin of cross contamination.	Time and qualified personnel are required in the analysis, indirect technique to detect gluten (does not quantify the presence of gluten).
**WESTERN BLOT**	Highly specific and sensitive, suitable for determining the gluten content in raw and processed foods.	Slow method, requires adequate training and specialization of analysts.
**MASS SPECTROMETRY (LC–MS)**	Speed, reproducibility, precision.	Complex instrumentation, expensive equipment, not a quantitative technique, etc.
**CHROMATOGRAPHY**	High capacity for the separation of different peptides.	Time consuming, difficult to automate for many samples.
**IMMUNOCHROMATOGRAPHIC STRIPS**	Very simple, fast method, visual interpretation.	It does not show the concentration of gluten in the sample.

**Table 3:** Gluten-specific methods commercially available worldwide. ELISA, Enzyme-Linked ImmunoSorbent Assay; PCR, polymerase chain reaction.

Format	Test Kit	Manufacturer	Target	Antibody
ELISA competitive	AgraQuant ELISA Gluten G12	Romer Labs	QPQLPY	G12 monoclonal
	RIDASCREEN Gliadin Competitive	R-Biopharm, AG	QQPFP, QQQFP, LQPFP, QLPFP	R5 monoclonal
	GlutenTox^®^ ELISA Competitive	Hygiena	QPQLPY	G12 monoclonal
ELISA sandwich	Veratox for Gliadin, 8480	Neogen Corp.	Gluten	USDA monoclonal
	Veratox for Gliadin R5, 8510	Neogen Corp.	QQPFP, QQQFP, LQPFP, QLPFP	R5 monoclonal
	MonoTrace Gluten ELISA Kit GLU-EK-96	BioFront Technologies	Gluten	Set of gluten-specific monoclonal antibodies
	RIDASCREEN^®^FAST Gliadin sensitive	R-Biopharm, AG	QQPFP, QQQFP, LQPFP, QLPFP	R5 monoclonal
	RIDASCREEN^®^FAST Gliadin	R-Biopharm, AG	QQPFP, QQQFP, LQPFP, QLPFP	R5 monoclonal
	AllergenControl ^TM^ Gluten Sandwich	Microbiologique Inc.	Gliadin	2D4
	Wheat Protein ELISA (MIoBS)	Morinaga Institute of Biological Sciences, Inc.	Gliadin	Polyclonal
	AllerTek Gluten	ELISA Technologies, Inc.	HMW glutenin	Skerritt monoclonal
	GlutenTox^®^ ELISA Rapid	Hygiena	QPQLPY	G12/A1 monoclonal
	Gluten-Check ELISA kit	Biocheck (UK)	QQPFP, QQQFP, LQPFP, QLPFP	R5 monoclonal
Lateral flow device (LFD)	AgraStrit Gluten G12	Romer Labs	QPQLPY	G12 monoclonal
	RIDA^®^QUICK Gliadin	R-Biopharm, AG	QQPFP, QQQFP, LQPFP, QLPFP	R5 monoclonal
	GlutenTox^®^ Sticks Plus	Hygiena	QPQLPY	G12/A1 monoclonal
	GlutenTox^®^ Pro	Hygiena	QPQLPY	G12/A1 monoclonal
	Nima Gluten sensor	Nima Labs, Inc.	Gluten	Nima antibody
	EZ Glutent^TM^	ELISA Technologies, Inc.	Gluten	anti-omega gliadin antibody
PCR test	SureFood^®^ ALLERGEN Gluten	R-Biopharm, AG	QQPFP, QQQFP, LQPFP, QLPFP	
	SureFood^®^ ALLERGEN 4plex Cereals	R-Biopharm, AG	QQPFP, QQQFP, LQPFP, QLPFP	

## References

[1] Taraghikhah N., Ashtari S., Asri N., Shahbazkhani B., Al-Dulaimi D., Rostami-Nejad M., Rezaei-Tavirani M., Razzaghi M.R., Zali M.R. (2020). An updated overview of spectrum of gluten-related disorders: Clinical and diagnostic aspects. BMC Gastroenterol..

[2] Ludvigsson J.F., Leffler D.A., Bai J.C., Biagi F., Fasano A., Green P.H., Hadjivassiliou M., Kaukinen K., Kelly C.P., Leonard J.N. (2013). The Oslo definitions for coeliac disease and related terms. Gut.

[3] Antiga E., Maglie R., Quintarelli L., Verdelli A., Bonciani D., Bonciolini V., Caproni M. (2019). Dermatitis herpetiformis: Novel perspectives. Front. Immunol..

[4] Rej A., Aziz I., Sanders D.S. (2020). Coeliac disease and noncoeliac wheat or gluten sensitivity. J. Intern. Med..

[5] Herrera M.J., Hermoso M.A., Quera R. (2009). An update on the pathogenesis of celiac disease. Rev. Med. Chile.

[6] Fasano A., Sapone A., Zevallos V., Schuppan D. (2015). Nonceliac gluten and wheat sensitivity. Gastroenterology.

[7] Caio G., Volta U., Sapone A., Leffler D.A., De Giorgio R., Catassi C., Fasano A. (2019). Celiac disease: A comprehensive current review. BMC Med..

[8] Lindfors K., Ciacci C., Kurppa K., Lundin K.E., Makharia G.K., Mearin M.L., Murray J.A., Verdu E.F., Kaukinen K. (2019). Coeliac disease. Nat. Rev. Dis. Primers.

[9] Hollon J.R., Cureton P.A., Martin M.L., Puppa E.L., Fasano A. (2013). Trace gluten contamination may play a role in mucosal and clinical recovery in a subgroup of diet-adherent non-response celiac disease patients. BMC Gastroenterol..

[10] Itzlinger A., Branchi F., Elli L., Schumann M. (2018). Gluten-free diet in celiac disease—Forever and for all?. Nutrients.

[11] Rosell C.M., Barro F., Sousa C., Mena M.C. (2014). Cereals for developing gluten-free products and analytical tools for gluten detection. J. Cereal Sci..

[12] Rai S., Kaur A., Chopra C.S. (2018). Gluten-free products for celiac susceptible people. Front. Nutr..

[13] Codex Standard 118-1979. http://www.fao.org/fao-who-codexalimentarius/sh-proxy/en/?lnk=1&url=https%253A%252F%252Fworkspace.fao.org%252Fsites%252Fcodex%252FStandards%252FCXS%2B118-1979%252FCXS_118e_2015.pdf.

[14] Francavilla R., Cristofori F., Stella M., Borrelli G., Naspi G., Castellaneta S. (2014). Treatment of celiac disease: From gluten-free diet to novel therapies. Minerva Pediatr..

[15] Leonard M.M., Cureton P., Fasano A. (2017). Indications and use of the gluten contamination elimination diet for patients with non-responsive celiac disease. Nutrients.

[16] Shehab D.I. (2013). Celiac disease. Egypt J. Intern. Med..

[17] Koerner T.B., Abbott M., Godefroy S.B., Popping B., Yeung J.M., Diaz-Amigo C., Roberts J., Taylor S.L., Baumert J.L., Ulberth F. (2013). Validation procedures for quantitative gluten ELISA methods: AOAC allergen community guidance and best practices. J. AOAC Int..

[18] Segura V., Díaz J., Ruiz-Carnicer Á., Muñoz-Suano A., Carrillo-Carrión C., Sousa C., Cebolla Á., Comino I. (2021). Rapid, Effective and Versatile Extraction of Gluten in Food with Application on Different Immunological Methods. Foods.

[19] Deutsch H., Poms R., Heeres H., van der Kamp J.W., Arendt E.K., dal Bello F. (2008). Labeling and regulatory issues. Gluten-Free Cereal Products and Beverages.

[20] McIntosh J., Flanagan A., Madden N., Mulcahy M., Dargan L., Walker M., Burns D.T. (2011). Awareness of coeliac disease and the gluten status of ‘gluten-free’ food obtained on request in catering outlets in Ireland. Int. J. Food Sci. Technol..

[21] Gibert A., Kruizinga A.G., Neuhold S., Houben G.F., Canela M.A., Fasano A., Catassi C. (2013). Might gluten traces in wheat substitutes pose a risk in patients with celiac disease? A population-based probabilistic approach to risk estimation. Am. J. Clin. Nutr..

[22] Lee H.J., Anderson Z., Ryu D. (2014). Gluten contamination in foods labeled as “gluten-free” in the United States. J. Food Prot..

[23] Oliveira O.M., Zandonadi R.P., Gandolfi L., de Almeida D.C., Almeida L.M., Pratesi R. (2014). Evaluation of the presence of gluten in beans served at self-service restaurants: A problem for celiac disease carriers. J. Culin. Sci. Technol..

[24] Sharma G.M., Pereira M., Williams K.M. (2015). Gluten detection in foods available in the United States—A market survey. Food Chem..

[25] Bustamante M.A., Fernandez-Gil M.P., Churruca I., Miranda J., Lasa A., Navarro V., Simon E. (2017). Evolution of gluten content in cereal-based gluten-free products: An overview from 1998 to 2016. Nutrients.

[26] Atasoy G., Gokhisar O.K., Turhan M. (2020). Gluten contamination in manufactured gluten-free food in Turkey. Food Addit. Contam. Part. A Chem. Anal. Control. Expo. Risk Assess..

[27] Bianchi D.M., Maurella C., Gallina S., Gorrasi I.S., Caramelli M., Decastelli L. (2018). Analysis of gluten content in gluten-free pizza from certified take-away pizza restaurants. Foods.

[28] Farage P., Zandonadi R.P., Gandolfi L., Pratesi R., Falcomer A.L., Arujo L.S., Yoshio Nakano E., Cortez-Ginani V. (2019). Accidental gluten contamination in traditional lunch meals from food services in Brasilia, Brazil. Nutrients.

[29] Lerner B.A., Phan Vo L.T., Yates S., Rundle A.G., Green P.H., Lebwohl B. (2019). Detection of gluten in gluten-free labeled restaurant food: Analysis of crowd-sourced data. Am. J. Gastroenterol..

[30] Raju N., Joshi A.K., Vahini R., Deepika T., Bhaskarachari K., Devindra S. (2020). Gluten contamination in labelled and naturally gluten-free grain products in Southern India. Food Addit. Contam. Part A Chem. Anal. Control. Expo. Risk Assess..

[31] Thompson T., Lee A.R., Grace T. (2010). Gluten contamination of grains, seeds, and flours in the United States: A pilot study. J. Am. Diet. Assoc..

[32] Koerner T.B., Cleroux C., Poirier C., Cantin I., La Vieille S., Hayward S., Dubois S. (2013). Gluten contamination of naturally gluten-free flours and starches used by Canadians with celiac disease. Food Addit. Contam. Part A Chem. Anal. Control. Expo. Risk Assess..

[33] Hassan H., Elaridi J., Bassil M. (2017). Evaluation of gluten in gluten-free-labeled foods and assessment of exposure level to gluten among celiac patients in Lebanon. Int. J. Food Sci. Nutr..

[34] Verma A.K., Gatti S., Galeazzi T., Monachesi C., Padella L., Baldo G.D., Annibali R., Lionetti E., Catassi C. (2017). Gluten contamination in naturally or labeled gluten-free products marketed in Italy. Nutrients.

[35] Farage P., de Medeiros Nobrega Y.K., Pratesi R., Gandolfi L., Assuncao P., Zandonadi R.P. (2017). Gluten contamination in gluten-free bakery products: A risk for coeliac disease patients. Public Health Nutr..

[36] Scherf K.A., Wieser H., Koehler P. (2016). Improved quantitation of gluten in wheat starch for celiac disease patients by gel-permeation high-performance liquid chromatography with fluorescence detection (GP-HPLC-FLD). J. Agric. Food Chem..

[37] Falcomer A.L., Santos-Araujo L., Farage P., Santos-Monteiro J., Yoshio-Nakano E., Puppin-Zandonadi R. (2018). Gluten contamination in food services and industry: A systematic review. Crit. Rev. Sci. Nutr..

[38] Weisbrod V.M., Silvester J.A., Raber C., Suslovic W., Coburn S.S., Raber B., McMahon J., Damast A., Kramer Z., Kerzner B. (2020). A quantitative assessment of gluten cross-contact in the school environment for children with celiac disease. J. Pediatr. Gastroenterol. Nutr..

[39] Cohen I., Day A.S., Shaoul R. (2019). To be or not to be? An update on the ongoing debate on oats for patients with celiac disease. Front. Pediatr..

[40] Hoffmanova I., Sanchez D., Szczepankova A., Tlaskalova-Hogenova H. (2019). The pros and cons of using oat in a gluten-free diet for celiac patients. Nutrients.

[41] Boison J., Allred L., Almy D., Anderson L., Baumert J., Bhandari S., Cebolla A., Chen Y., Crowley E., Diaz-Amigo C. (2018). Standard method performance requirements (SMPRs^®^) 2017.21: Quantitation of wheat, rye and barley gluten in oats. J. AOAC.

[42] Chen Y., Fritz R.D., Kock L., Garg D., Davis R.M., Kasturi P. (2018). A stepwise test-all-positives methodology to assess gluten-kernel contamination at the serving-size level in gluten-free (GF) oat production. Food Chem..

[43] Hernando A., Mujico J.R., Juanas D., Méndez E. (2006). Confirmation of the cereal type in oat products highly contaminated with gluten. J. Am. Diet. Assoc..

[44] Koerner T.B., Cléroux C., Poirier C., Cantin I., Alimkulov A., Elamparo H. (2011). Gluten contamination in the Canadian commercial oat supply. Food Addit. Contam. Part A Chem. Anal. Control. Expo. Risk Assess..

[45] Silano M., Dessì M., De Vincenzi M., Cornell H. (2007). In Vitro tests indicate that certain varieties of oats may be harmful to patients with coeliac disease. J Gastroenterol. Hepatol..

[46] Silano M., Di Benedetto R., Maialetti F., De Vincenzi A., Calcaterra R., Cornell H.J., De Vincenzi M. (2007). Avenins from different cultivars of oats elicit response by coeliac peripheral lymphocytes. Scand. J. Gastroenterol..

[47] Comino I., Real A., de Lorenzo L., Cornell H., López-Casado M.Á., Barro F., Lorite P., Torres M.I., Cebolla A., Sousa C. (2011). Diversity in oat potential immunogenicity: Basis for the selection of oat varieties with no toxicity in coeliac disease. Gut.

[48] Silano M., Pozo E.P., Uberti F., Manferdelli S., Del Pinto T., Felli C., Budelli A., Vincentini O., Restani P. (2014). Diversity of oat varieties in eliciting the early inflammatory events in celiac disease. Eur. J. Nutr..

[49] Mujico J.R., Mitea C., Gilissen L.J., de Ru A., van Veelen P., Smulders M.J., Koning F. (2011). Natural variation in avenin epitopes among oat varieties: Implications for celiac disease. J. Cereal Sci..

[50] Benoit L., Masiri J., Del Blanco I.A., Meshgi M., Gendel S.M., Samadpour M. (2017). Assessment of Avenins from Different Oat Varieties Using R5-Based Sandwich ELISA. J. Agric. Food Chem..

[51] Gimenez M.J., Real A., Garcia-Molina M.D., Sousa C., Barro F. (2017). Characterization of celiac disease related oat proteins: Bases for the development of high quality oat varieties suitable for celiac patients. Sci. Rep..

[52] Arentz-Hansen H., Fleckenstein B., Molberg Ø., Scott H., Koning F., Jung G., Roepstorff P., Lundin K.E., Sollid L.M. (2004). The molecular basis for oat intolerance in patients with celiac disease. PLoS Med..

[53] Real A., Comino I., de Lorenzo L., Merchán F., Gil-Humanes J., Giménez M.J., López-Casado M.Á., Torres M.I., Cebolla Á., Sousa C. (2012). Molecular and immunological characterization of gluten proteins isolated from oat cultivars that differ in toxicity for celiac disease. PLoS ONE.

[54] Comino I., Bernardo D., Bancel E., Moreno M.L., Sánchez B., Barro F., Šuligoj T., Ciclitira P.J., Cebolla Á., Knight S.C. (2016). Identification and molecular characterization of oat peptides implicated on coeliac immune response. Food Nutr. Res..

[55] Wieser H., Koehler P., Scherf K.A. (2020). Wheat—An Exceptional Cro.

[56] Shah A.V., Serajuddin A.T., Mangione R.A. (2018). Making all medications gluten free. J. Pharm. Sci..

[57] Verma A.K., Lionetti E., Gatti S., Franceschini E., Catassi G.N., Catassi C. (2019). Contribution of oral hygiene and cosmetics on contamination of gluten-free diet: Do celiac customers need to worry about?. J. Pediatr. Gastroenterol. Nutr..

[58] Allred L.K., Park E.S. (2012). EZ Gluten for the qualitative detection of gluten in foods, beverages, and environmental surfaces. J. AOAC Int..

[59] Zhang J., Portela S.B., Horrell J.B., Leung A., Weitmann D.R., Artiuch J.B., Wilson S.M., Cipriani M., Slakey L.K., Burt A.M. (2019). An integrated, accurate, rapid and economical handheld consumer gluten detector. Food Chem..

[60] Bruins Slot I.D., Bremer M.G., van der Fels-Klerx I., Hamer R.J. (2015). Evaluating the performance of gluten ELISA test kits: The numbers do not tell the tale. Cereal Chem..

[61] Mena M.C., Sousa C., Arranz E., Fernández-Bañares F., Rosell C.M., Rodrigo L., Peña A.S. (2015). Analytical tools for gluten detection, Policies and regulation. Advances in the Understanding of Gluten Related Pathology and the Evolution of Gluten-Free Foods.

[62] Rzychon M., Brohée M., Cordeiro F., Haraszi R., Ulberth F., O’Connor G. (2017). The feasibility of harmonizing gluten ELISA measurements. Food Chem..

[63] Lexhaller B., Tompos C., Scherf K.A. (2016). Comparative analysis of prolamin and glutelin fractions from wheat, rye, and barley with five sandwich ELISA test kits. Anal. Bioanal. Chem..

[64] Lexhaller B., Tompos C., Scherf K.A. (2017). Fundamental study on reactivities of gluten protein types from wheat, rye and barley with five sandwich ELISA test kits. Food Chem..

[65] Schopf M., Scherf K.A. (2018). Wheat cultivar and species influence variability of gluten ELISA analyses based on polyclonal and monoclonal antibodies R5 and G12. J. Cereal Sci..

[66] Rallabhandi P., Sharma G.M., Pereira M., Williams K.M. (2015). Immunological characterization of the gluten fractions and their hydrolysates from wheat, rye and barley. J. Agric. Food Chem..

[67] Frazer A.C., Fletcher R.F., Ross C.A., Shaw B., Sammons H.G., Schneider R. (1959). Gluten-induced enteropathy: The effect of partially digested gluten. Lancet.

[68] Picariello G., Mamone G., Cutignano A., Fontana A., Zurlo L., Addeo F., Ferranti P. (2015). Proteomics, peptidomics, and immunogenic potential of wheat beer (weissbier). J. Agric. Food Chem..

[69] Real A., Comino I., Moreno M.D.L., López-Casado M.Á., Lorite P., Isabel Torres M., Cebolla Á., Sousa C. (2014). Identification and in vitro reactivity of celiac immunoactive peptides in an apparent gluten-free beer. PLoS ONE.

[70] Wei G., Tian N., Siezen R., Schuppan D., Helmerhorst E.J. (2016). Identification of food-grade subtilisins as gluten-degrading enzymes to treat celiac disease. Am. J. Physiol. Gastrointest. Liver Physiol..

[71] Spaenij-Dekking L., Kooy-Winkelaar Y., Van Veelen P., Drijfhout J.W., Jonker H., Van Soest L., Smulders M.J.M., Bosch D., Gilissen L.J.W.J., Koning F. (2005). Natural variation in toxicity of wheat: Potential for selection of nontoxic varieties for celiac disease patients. Gastroenterology.

[72] Gregorini A., Colomba M., Julia Ellis H., Ciclitira P.J. (2009). Immunogenicity characterization of two ancient wheat α-gliadin peptides related to coeliac disease. Nutrients.

[73] Schalk K., Lexhaller B., Koehler P., Scherf K.A. (2017). Isolation and characterization of gluten protein types from wheat, rye, barley and oats for use as reference materials. PLoS ONE.

[74] Mamone G., Ferranti P., Rossi M., Roepstorff P., Fierro O., Malorni A., Addeo F. (2007). Identification of a peptide from alpha-gliadin resistant to digestive enzymes: Implications for celiac disease. J. Chromatogr. B Analyt. Technol. Biomed. Life Sci..

[75] Comino I., Real A., Gil-Humanes J., Pistón F., de Lorenzo L., Moreno M.L., López-Casado M.Á., Lorite P., Cebolla A., Torres M.I. (2012). Significant differences in coeliac immunotoxicity of barley varieties. Mol. Nutr. Food Res..

[76] Wieser H., Koehler P. (2009). Is the calculation of the gluten content by multiplying the prolamin content by a factor of 2 valid?. Eur. Food Res. Technol..

[77] Scherf K.A. (2017). Gluten analysis of wheat starches with seven commercial ELISA test kits—Up to six different values. Food Anal. Methods.

